# 14-Hy­droxy-11-[(*E*)-4-meth­oxy­benzyl­idene]-8-(4-meth­oxy­phen­yl)-5-thia-3,13-diaza­hepta­cyclo­[13.7.1.1^9,13^.0^2,9^.0^2,14^.0^3,7^.0^19,23^]tetra­cosa-1(22),15(23),16,18,20-pentaen-10-one

**DOI:** 10.1107/S160053681104061X

**Published:** 2011-10-08

**Authors:** Raju Suresh Kumar, Hasnah Osman, A. S. Abdul Rahim, Madhukar Hemamalini, Hoong-Kun Fun

**Affiliations:** a School of Chemical Sciences, Universiti Sains Malaysia, 11800 USM, Penang, Malaysia; b School of Pharmaceutical Sciences, Universiti Sains Malaysia, 11800 USM, Penang, Malaysia; c X-ray Crystallography Unit, School of Physics, Universiti Sains Malaysia, 11800 USM, Penang, Malaysia

## Abstract

In the title compound, C_36_H_32_N_2_O_4_S, the piperidine ring adopts a chair conformation, while the five-membered pyrrolidine (with a C atom as the flap atom) and thia­zolidine (with the S atom as the flap atom) rings adopt envelope conformations. The naphthalene ring system makes dihedral angles of 18.82 (5) and 40.92 (5)° with the two meth­oxy-substituted benzene rings. In the crystal, centrosymmetrically-related mol­ecules are linked into dimers *via* pairs of C—H⋯O and C—H⋯N hydrogen bonds. An intra­molecular O—H⋯N hydrogen bond is also observed. The crystal structure is further stabilized by C—H⋯π inter­actions.

## Related literature

For details of cyclo­addition, see: Tsuge & Kanemasa (1989 ▶); Nair & Suja (2007 ▶); Aicher *et al.* (1998 ▶); Lalezari & Schwartz (1988 ▶). For ring conformations, see: Cremer & Pople (1975 ▶). For the stability of the temperature controller used in the data collection, see: Cosier & Glazer (1986 ▶).
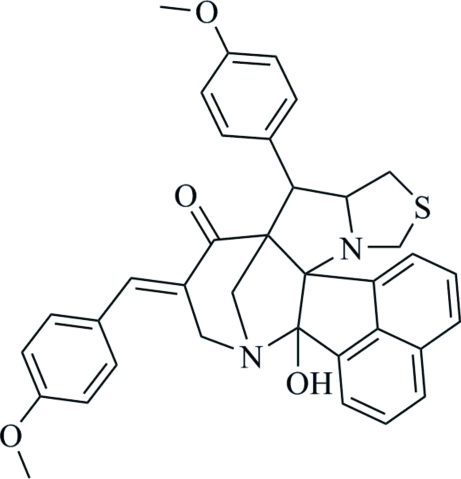


## Experimental

### 

#### Crystal data

C_36_H_32_N_2_O_4_S
*M* *_r_* = 588.70
Triclinic, 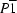

*a* = 10.6287 (1) Å
*b* = 11.8672 (2) Å
*c* = 12.6588 (2) Å
α = 84.439 (1)°
β = 75.105 (1)°
γ = 68.553 (1)°
*V* = 1436.19 (4) Å^3^
*Z* = 2
Mo *K*α radiation
μ = 0.16 mm^−1^
*T* = 100 K
0.37 × 0.25 × 0.16 mm

#### Data collection

Bruker SMART APEXII CCD diffractometer
Absorption correction: multi-scan (*SADABS*; Bruker, 2009 ▶) *T*_min_ = 0.943, *T*_max_ = 0.975
39492 measured reflections
11591 independent reflections
9231 reflections with *I* > 2σ(*I*)
*R*_int_ = 0.036

#### Refinement

*R*[*F* ^2^ > 2σ(*F* ^2^)] = 0.050
*wR*(*F* ^2^) = 0.142
*S* = 1.02
11591 reflections
394 parameters
H atoms treated by a mixture of independent and constrained refinement
Δρ_max_ = 0.66 e Å^−3^
Δρ_min_ = −0.38 e Å^−3^

### 

Data collection: *APEX2* (Bruker, 2009 ▶); cell refinement: *SAINT* (Bruker, 2009 ▶); data reduction: *SAINT*; program(s) used to solve structure: *SHELXTL* (Sheldrick, 2008 ▶); program(s) used to refine structure: *SHELXTL*; molecular graphics: *SHELXTL*; software used to prepare material for publication: *SHELXTL* and *PLATON* (Spek, 2009 ▶).

## Supplementary Material

Crystal structure: contains datablock(s) global, I. DOI: 10.1107/S160053681104061X/hb6431sup1.cif

Structure factors: contains datablock(s) I. DOI: 10.1107/S160053681104061X/hb6431Isup2.hkl

Supplementary material file. DOI: 10.1107/S160053681104061X/hb6431Isup3.cml

Additional supplementary materials:  crystallographic information; 3D view; checkCIF report

## Figures and Tables

**Table 1:** Hydrogen-bond geometry (Å, °) *Cg*7 and *Cg*9 centroids of the C20–C25 and C31–C36 rings, respectively.

*D*—H⋯*A*	*D*—H	H⋯*A*	*D*⋯*A*	*D*—H⋯*A*
O1—H1*O*1⋯N2	0.86 (2)	1.96 (2)	2.6253 (14)	133.3 (19)
C24—H24*A*⋯N1^i^	0.95	2.61	3.4447 (15)	146
C26—H26*C*⋯O1^i^	0.98	2.48	3.4078 (17)	157
C19—H19*B*⋯*Cg*7^ii^	0.98	2.82	3.4652 (18)	124
C26—H26*B*⋯*Cg*7^iii^	0.98	2.87	3.7720 (14)	154
C9—H9*B*⋯*Cg*9^iv^	0.99	2.87	3.8211 (14)	161

Symmetry codes: (i) 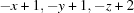; (ii) 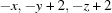; (iii) 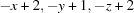; (iv) 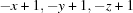.

**Fig. 1.**
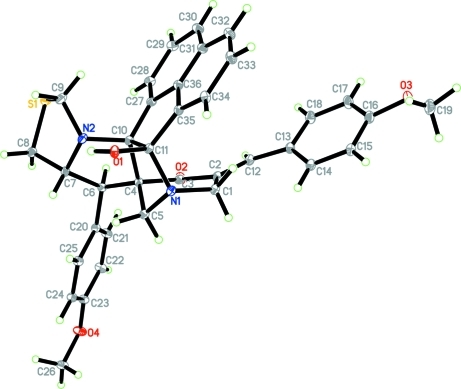
The asymmetric unit of the title compound, showing 30% probability displacement ellipsoids.

**Fig. 2.**
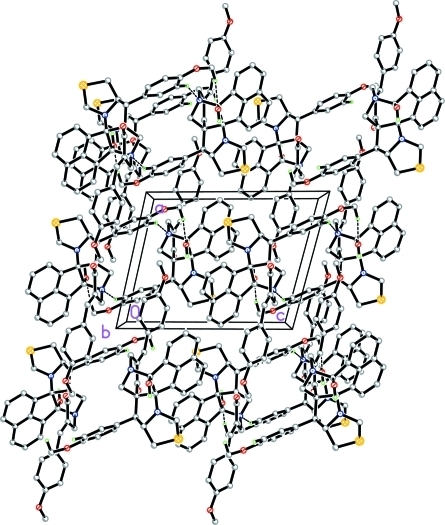
The crystal packing of the title compound (I). H atoms not involved in hydrogen bonding are omitted.

## supplementary crystallographic information

### Comment

Three-component reactions involving [3+2]-cycloaddition of azomethine ylides to olefinic dipolarophiles constitutes a facile approach for the construction of five membered heterocyclic rings of biological importance (Tsuge & Kanemasa, 1989; Nair & Suja, 2007). Among these heterocycles, pyrrolo[2,1-b]thiazole is an unusual ring system with antineoplastic (Lalezari & Schwartz, 1988) and hypoglycemic (Aicher *et al.*, 1998) activities. In this paper we wish to report the crystal structure determination of the title compound possessing the biologically-active pyrrolothiazole ring.

The asymmetric unit of the title compound is shown in Fig. 1. The six-membered piperidine (N1/C1–C5) ring adopts a chair conformation [Q = 0.6172 (12) Å; θ = 138.18 (11)° and φ = 119.73 (17)°; Cremer & Pople, 1975] while the five-membered pyrrolidine (N2/C4/C6,C7/C10) and thiazolidine (S1/N2/C7–C9) rings adopt an envelope conformation with the C6 (displacement -0.217 (1) Å) and the S1 (displacement 0.284 (1) Å) atoms as the flap atoms and with puckering parameters, Q = 0.3565 (13) Å; φ = 89.37 (19)°; and Q = 0.5087 (11) Å; and  φ = 0.44 (14)° repectively. The naphthalene (C27–C36) ring makes dihedral angles of 18.82 (5)° and 40.92 (5)° with the two methoxy-substituted (C13–C18)/(C20–C25) phenyl rings.

In the crystal structure, (Fig. 2), the centrosymmetrically-related molecules are linked into dimers *via* pairs of intermolecular C—H···O and C—H···N (Table 1) hydrogen bonds. An intramolecular O—H..N hydrogen bond is also observed.  The crystal structure is further stabilized by weak C—H···π interactions involving the centroids Cg7 and Cg9  of the (C20–C25) and (C31–C36) rings, respectively.

### Experimental

A mixture of 3,5-bis[(*E*)-(4-methoxyphenyl) methylidene]-tetrahydro-4(1*H*)-pyridinone (1 mmol), acenaphthenequinone (1 mmol), and thiazolidine-4-carboxylic acid (1 mmol) were dissolved in methanol (5 mL) and refluxed for 1 hour. After completion of the reaction as evident from TLC, the mixture was poured into water (50 mL). The precipitated solid was filtered and washed with water to obtain the product which was further purified by recrystallisation from pet.ether-ethylacetate mixture to yield colourless blocks of (I).

### Refinement

Atom H1O1  was located from a difference Fourier maps and refined freely [O–H = 0.86 (2) Å]. The remaining H atoms were positioned geometrically [C–H = 0.95–1.00 Å] and were refined using a riding model, with *U*_iso_(H) = 1.2 or 1.5 *U*_eq_(C). A rotating group model was applied to the methyl groups.

### Crystal data

**Table:**

C_36_H_32_N_2_O_4_S	*Z* = 2
*M**_r_* = 588.70	*F*(000) = 620
Triclinic, *P*1	*D*_x_ = 1.361 Mg m^−^^3^
Hall symbol: -P 1	Mo *K*α radiation, λ = 0.71073 Å
*a* = 10.6287 (1) Å	Cell parameters from 9888 reflections
*b* = 11.8672 (2) Å	θ = 2.3–35.1°
*c* = 12.6588 (2) Å	µ = 0.16 mm^−^^1^
α = 84.439 (1)°	*T* = 100 K
β = 75.105 (1)°	Block, colourless
γ = 68.553 (1)°	0.37 × 0.25 × 0.16 mm
*V* = 1436.19 (4) Å^3^	

### Data collection

**Table:**

Bruker SMART APEXII CCD diffractometer	11591 independent reflections
Radiation source: fine-focus sealed tube	9231 reflections with *I* > 2σ(*I*)
graphite	*R*_int_ = 0.036
φ and ω scans	θ_max_ = 34.0°, θ_min_ = 1.7°
Absorption correction: multi-scan (*SADABS*; Bruker, 2009)	*h* = −13→16
*T*_min_ = 0.943, *T*_max_ = 0.975	*k* = −18→18
39492 measured reflections	*l* = −19→19

### Refinement

**Table:**

Refinement on *F*^2^	Primary atom site location: structure-invariant direct methods
Least-squares matrix: full	Secondary atom site location: difference Fourier map
*R*[*F*^2^ > 2σ(*F*^2^)] = 0.050	Hydrogen site location: inferred from neighbouring sites
*wR*(*F*^2^) = 0.142	H atoms treated by a mixture of independent and constrained refinement
*S* = 1.02	*w* = 1/[σ^2^(*F*_o_^2^) + (0.0751*P*)^2^ + 0.5144*P*] where *P* = (*F*_o_^2^ + 2*F*_c_^2^)/3
11591 reflections	(Δ/σ)_max_ = 0.001
394 parameters	Δρ_max_ = 0.66 e Å^−^^3^
0 restraints	Δρ_min_ = −0.38 e Å^−^^3^

### Special details

**Table:**

Experimental. The crystal was placed in the cold stream of an Oxford Cryosystems Cobra open-flow nitrogen cryostat (Cosier & Glazer, 1986) operating at 100.0 (1) K.
Geometry. All s.u.'s (except the s.u. in the dihedral angle between two l.s. planes) are estimated using the full covariance matrix. The cell s.u.'s are taken into account individually in the estimation of s.u.'s in distances, angles and torsion angles; correlations between s.u.'s in cell parameters are only used when they are defined by crystal symmetry. An approximate (isotropic) treatment of cell s.u.'s is used for estimating s.u.'s involving l.s. planes.
Refinement. Refinement of F^2^ against ALL reflections. The weighted R-factor wR and goodness of fit S are based on F^2^, conventional R-factors R are based on F, with F set to zero for negative F^2^. The threshold expression of F^2^ > 2σ(F^2^) is used only for calculating R-factors(gt) etc. and is not relevant to the choice of reflections for refinement. R-factors based on F^2^ are statistically about twice as large as those based on F, and R- factors based on ALL data will be even larger.

### Fractional atomic coordinates and isotropic or equivalent isotropic displacement parameters (Å^2^)

**Table:**

	*x*	*y*	*z*	*U*_iso_*/*U*_eq_	
S1	0.79522 (3)	0.60158 (3)	0.48963 (2)	0.02192 (7)	
O1	0.36989 (8)	0.50498 (7)	0.72474 (7)	0.01646 (15)	
O2	0.46670 (9)	0.92470 (8)	0.78875 (8)	0.02136 (17)	
O3	−0.38126 (10)	1.24757 (10)	0.79511 (9)	0.0306 (2)	
O4	0.89106 (10)	0.68108 (9)	1.10873 (8)	0.02435 (19)	
N1	0.28341 (9)	0.66632 (8)	0.84751 (8)	0.01489 (16)	
N2	0.58835 (9)	0.56730 (9)	0.64184 (8)	0.01619 (17)	
C1	0.18792 (11)	0.79213 (10)	0.87130 (9)	0.01641 (19)	
H1A	0.1511	0.8009	0.9515	0.020*	
H1B	0.1081	0.8072	0.8385	0.020*	
C2	0.25141 (11)	0.88926 (10)	0.83004 (9)	0.01574 (18)	
C3	0.40668 (11)	0.85241 (10)	0.80088 (9)	0.01529 (18)	
C4	0.48625 (10)	0.71733 (10)	0.78310 (9)	0.01380 (17)	
C5	0.41421 (11)	0.65015 (10)	0.87649 (9)	0.01572 (18)	
H5A	0.4719	0.5633	0.8785	0.019*	
H5B	0.3961	0.6867	0.9484	0.019*	
C6	0.64462 (11)	0.67997 (10)	0.75962 (9)	0.01479 (18)	
H6A	0.6715	0.7393	0.7055	0.018*	
C7	0.69662 (11)	0.55899 (10)	0.69999 (9)	0.01564 (18)	
H7A	0.7041	0.4916	0.7547	0.019*	
C8	0.83679 (11)	0.53339 (11)	0.61614 (10)	0.0188 (2)	
H8A	0.8876	0.4451	0.6070	0.023*	
H8B	0.8953	0.5696	0.6400	0.023*	
C9	0.64853 (12)	0.54767 (12)	0.52515 (10)	0.0212 (2)	
H9A	0.5784	0.5931	0.4833	0.025*	
H9B	0.6810	0.4605	0.5078	0.025*	
C10	0.46097 (10)	0.67354 (10)	0.68033 (9)	0.01421 (17)	
C11	0.32921 (10)	0.63151 (10)	0.73099 (9)	0.01408 (17)	
C12	0.17995 (11)	1.00541 (10)	0.80811 (10)	0.01742 (19)	
H12A	0.2345	1.0553	0.7851	0.021*	
C13	0.03109 (11)	1.06591 (10)	0.81448 (10)	0.01782 (19)	
C14	−0.07494 (12)	1.02983 (10)	0.88129 (10)	0.0185 (2)	
H14A	−0.0513	0.9639	0.9303	0.022*	
C15	−0.21409 (12)	1.08765 (11)	0.87811 (10)	0.0202 (2)	
H15A	−0.2838	1.0608	0.9239	0.024*	
C16	−0.24999 (12)	1.18508 (11)	0.80725 (11)	0.0225 (2)	
C17	−0.14648 (13)	1.22501 (12)	0.74238 (12)	0.0259 (2)	
H17A	−0.1708	1.2925	0.6951	0.031*	
C18	−0.00926 (13)	1.16714 (11)	0.74650 (11)	0.0234 (2)	
H18A	0.0595	1.1963	0.7025	0.028*	
C19	−0.48594 (14)	1.19644 (15)	0.84307 (14)	0.0335 (3)	
H19A	−0.5714	1.2426	0.8192	0.050*	
H19B	−0.5051	1.1997	0.9229	0.050*	
H19C	−0.4530	1.1120	0.8199	0.050*	
C20	0.70703 (11)	0.67646 (10)	0.85510 (9)	0.01501 (18)	
C21	0.75256 (12)	0.76997 (11)	0.86841 (10)	0.0190 (2)	
H21A	0.7418	0.8356	0.8180	0.023*	
C22	0.81297 (13)	0.76854 (11)	0.95352 (11)	0.0215 (2)	
H22A	0.8425	0.8332	0.9612	0.026*	
C23	0.83076 (12)	0.67281 (11)	1.02806 (9)	0.0180 (2)	
C24	0.78598 (12)	0.57861 (10)	1.01762 (9)	0.01754 (19)	
H24A	0.7968	0.5132	1.0683	0.021*	
C25	0.72497 (12)	0.58202 (10)	0.93148 (9)	0.01717 (19)	
H25A	0.6945	0.5178	0.9245	0.021*	
C26	0.90991 (13)	0.58491 (12)	1.18668 (10)	0.0228 (2)	
H26A	0.9547	0.6000	1.2397	0.034*	
H26B	0.9688	0.5083	1.1490	0.034*	
H26C	0.8192	0.5803	1.2248	0.034*	
C27	0.41283 (11)	0.76642 (10)	0.59441 (9)	0.01534 (18)	
C28	0.47477 (12)	0.83835 (11)	0.52540 (10)	0.0191 (2)	
H28A	0.5635	0.8372	0.5288	0.023*	
C29	0.40394 (13)	0.91443 (11)	0.44894 (10)	0.0217 (2)	
H29A	0.4476	0.9630	0.4006	0.026*	
C30	0.27418 (13)	0.92003 (11)	0.44276 (10)	0.0217 (2)	
H30A	0.2301	0.9716	0.3906	0.026*	
C31	0.20612 (12)	0.84865 (10)	0.51443 (10)	0.0183 (2)	
C32	0.07319 (12)	0.84443 (11)	0.51840 (11)	0.0217 (2)	
H32A	0.0199	0.8934	0.4700	0.026*	
C33	0.02096 (12)	0.76951 (11)	0.59229 (11)	0.0216 (2)	
H33A	−0.0680	0.7678	0.5933	0.026*	
C34	0.09598 (11)	0.69493 (10)	0.66679 (10)	0.0186 (2)	
H34A	0.0577	0.6446	0.7174	0.022*	
C35	0.22508 (11)	0.69716 (10)	0.66413 (9)	0.01528 (18)	
C36	0.27902 (11)	0.77326 (10)	0.58842 (9)	0.01567 (18)	
H1O1	0.459 (2)	0.4815 (19)	0.7015 (17)	0.038 (5)*	

### Atomic displacement parameters (Å^2^)

**Table:**

	*U*^11^	*U*^22^	*U*^33^	*U*^12^	*U*^13^	*U*^23^
S1	0.01679 (12)	0.03083 (16)	0.01653 (13)	−0.00899 (11)	−0.00024 (9)	−0.00062 (11)
O1	0.0143 (3)	0.0127 (3)	0.0219 (4)	−0.0046 (3)	−0.0028 (3)	−0.0023 (3)
O2	0.0192 (4)	0.0174 (4)	0.0302 (5)	−0.0093 (3)	−0.0059 (3)	−0.0015 (3)
O3	0.0181 (4)	0.0280 (5)	0.0396 (6)	−0.0001 (3)	−0.0079 (4)	−0.0013 (4)
O4	0.0323 (5)	0.0277 (5)	0.0232 (4)	−0.0175 (4)	−0.0158 (4)	0.0060 (4)
N1	0.0150 (4)	0.0148 (4)	0.0147 (4)	−0.0059 (3)	−0.0015 (3)	−0.0021 (3)
N2	0.0135 (4)	0.0190 (4)	0.0157 (4)	−0.0057 (3)	−0.0012 (3)	−0.0052 (3)
C1	0.0153 (4)	0.0151 (4)	0.0181 (5)	−0.0059 (3)	−0.0004 (3)	−0.0037 (4)
C2	0.0151 (4)	0.0160 (5)	0.0164 (5)	−0.0064 (3)	−0.0016 (3)	−0.0035 (4)
C3	0.0161 (4)	0.0157 (4)	0.0149 (4)	−0.0065 (3)	−0.0031 (3)	−0.0026 (4)
C4	0.0138 (4)	0.0144 (4)	0.0140 (4)	−0.0060 (3)	−0.0030 (3)	−0.0010 (3)
C5	0.0155 (4)	0.0162 (4)	0.0158 (4)	−0.0065 (3)	−0.0026 (3)	−0.0007 (4)
C6	0.0139 (4)	0.0156 (4)	0.0153 (4)	−0.0061 (3)	−0.0030 (3)	−0.0004 (3)
C7	0.0132 (4)	0.0163 (5)	0.0166 (5)	−0.0047 (3)	−0.0025 (3)	−0.0017 (4)
C8	0.0143 (4)	0.0213 (5)	0.0193 (5)	−0.0054 (4)	−0.0023 (4)	−0.0022 (4)
C9	0.0175 (5)	0.0289 (6)	0.0174 (5)	−0.0085 (4)	−0.0010 (4)	−0.0085 (4)
C10	0.0131 (4)	0.0146 (4)	0.0151 (4)	−0.0056 (3)	−0.0021 (3)	−0.0017 (3)
C11	0.0140 (4)	0.0135 (4)	0.0154 (4)	−0.0059 (3)	−0.0024 (3)	−0.0017 (3)
C12	0.0167 (4)	0.0153 (5)	0.0203 (5)	−0.0065 (4)	−0.0027 (4)	−0.0020 (4)
C13	0.0170 (4)	0.0148 (5)	0.0209 (5)	−0.0051 (4)	−0.0032 (4)	−0.0025 (4)
C14	0.0176 (4)	0.0153 (5)	0.0213 (5)	−0.0051 (4)	−0.0028 (4)	−0.0026 (4)
C15	0.0164 (4)	0.0188 (5)	0.0238 (5)	−0.0052 (4)	−0.0023 (4)	−0.0041 (4)
C16	0.0190 (5)	0.0184 (5)	0.0269 (6)	−0.0016 (4)	−0.0053 (4)	−0.0050 (4)
C17	0.0232 (5)	0.0170 (5)	0.0318 (7)	−0.0023 (4)	−0.0049 (5)	0.0022 (5)
C18	0.0218 (5)	0.0162 (5)	0.0293 (6)	−0.0057 (4)	−0.0034 (4)	0.0019 (4)
C19	0.0169 (5)	0.0400 (8)	0.0387 (8)	−0.0043 (5)	−0.0039 (5)	−0.0079 (6)
C20	0.0142 (4)	0.0163 (4)	0.0155 (4)	−0.0066 (3)	−0.0034 (3)	0.0003 (4)
C21	0.0216 (5)	0.0188 (5)	0.0210 (5)	−0.0113 (4)	−0.0083 (4)	0.0043 (4)
C22	0.0268 (5)	0.0209 (5)	0.0245 (6)	−0.0145 (4)	−0.0119 (4)	0.0047 (4)
C23	0.0183 (4)	0.0210 (5)	0.0177 (5)	−0.0093 (4)	−0.0067 (4)	0.0015 (4)
C24	0.0194 (5)	0.0177 (5)	0.0169 (5)	−0.0082 (4)	−0.0052 (4)	0.0022 (4)
C25	0.0191 (4)	0.0168 (5)	0.0178 (5)	−0.0088 (4)	−0.0049 (4)	0.0010 (4)
C26	0.0241 (5)	0.0287 (6)	0.0194 (5)	−0.0121 (5)	−0.0093 (4)	0.0050 (4)
C27	0.0166 (4)	0.0165 (5)	0.0145 (4)	−0.0076 (4)	−0.0034 (3)	−0.0013 (4)
C28	0.0210 (5)	0.0208 (5)	0.0183 (5)	−0.0113 (4)	−0.0039 (4)	0.0005 (4)
C29	0.0256 (5)	0.0209 (5)	0.0207 (5)	−0.0116 (4)	−0.0055 (4)	0.0030 (4)
C30	0.0251 (5)	0.0198 (5)	0.0212 (5)	−0.0081 (4)	−0.0082 (4)	0.0036 (4)
C31	0.0195 (5)	0.0165 (5)	0.0198 (5)	−0.0061 (4)	−0.0069 (4)	0.0003 (4)
C32	0.0204 (5)	0.0195 (5)	0.0263 (6)	−0.0055 (4)	−0.0103 (4)	0.0010 (4)
C33	0.0166 (5)	0.0205 (5)	0.0291 (6)	−0.0063 (4)	−0.0082 (4)	−0.0007 (4)
C34	0.0155 (4)	0.0169 (5)	0.0238 (5)	−0.0065 (4)	−0.0046 (4)	−0.0005 (4)
C35	0.0146 (4)	0.0144 (4)	0.0171 (5)	−0.0055 (3)	−0.0029 (3)	−0.0021 (4)
C36	0.0161 (4)	0.0151 (4)	0.0167 (5)	−0.0061 (3)	−0.0040 (3)	−0.0017 (4)

### Geometric parameters (Å, °)

**Table:**

S1—C8	1.8072 (12)	C14—C15	1.3939 (16)
S1—C9	1.8318 (12)	C14—H14A	0.9500
O1—C11	1.4066 (13)	C15—C16	1.3927 (18)
O1—H1O1	0.86 (2)	C15—H15A	0.9500
O2—C3	1.2209 (13)	C16—C17	1.3966 (19)
O3—C16	1.3608 (15)	C17—C18	1.3789 (18)
O3—C19	1.4317 (19)	C17—H17A	0.9500
O4—C23	1.3657 (14)	C18—H18A	0.9500
O4—C26	1.4256 (15)	C19—H19A	0.9800
N1—C5	1.4689 (14)	C19—H19B	0.9800
N1—C1	1.4737 (14)	C19—H19C	0.9800
N1—C11	1.4783 (14)	C20—C25	1.3985 (15)
N2—C9	1.4552 (15)	C20—C21	1.4018 (15)
N2—C10	1.4805 (14)	C21—C22	1.3847 (17)
N2—C7	1.4879 (14)	C21—H21A	0.9500
C1—C2	1.5257 (15)	C22—C23	1.3941 (16)
C1—H1A	0.9900	C22—H22A	0.9500
C1—H1B	0.9900	C23—C24	1.3943 (16)
C2—C12	1.3499 (16)	C24—C25	1.3953 (16)
C2—C3	1.4971 (15)	C24—H24A	0.9500
C3—C4	1.5219 (15)	C25—H25A	0.9500
C4—C6	1.5300 (14)	C26—H26A	0.9800
C4—C5	1.5549 (14)	C26—H26B	0.9800
C4—C10	1.5645 (15)	C26—H26C	0.9800
C5—H5A	0.9900	C27—C28	1.3768 (15)
C5—H5B	0.9900	C27—C36	1.4159 (15)
C6—C20	1.5115 (15)	C28—C29	1.4237 (17)
C6—C7	1.5336 (16)	C28—H28A	0.9500
C6—H6A	1.0000	C29—C30	1.3785 (17)
C7—C8	1.5352 (15)	C29—H29A	0.9500
C7—H7A	1.0000	C30—C31	1.4233 (16)
C8—H8A	0.9900	C30—H30A	0.9500
C8—H8B	0.9900	C31—C36	1.4077 (16)
C9—H9A	0.9900	C31—C32	1.4199 (16)
C9—H9B	0.9900	C32—C33	1.3806 (17)
C10—C27	1.5169 (15)	C32—H32A	0.9500
C10—C11	1.6150 (14)	C33—C34	1.4204 (17)
C11—C35	1.5071 (15)	C33—H33A	0.9500
C12—C13	1.4629 (15)	C34—C35	1.3738 (15)
C12—H12A	0.9500	C34—H34A	0.9500
C13—C14	1.4005 (16)	C35—C36	1.4111 (15)
C13—C18	1.4072 (17)		
			
C8—S1—C9	86.85 (5)	C15—C14—C13	122.04 (11)
C11—O1—H1O1	103.5 (14)	C15—C14—H14A	119.0
C16—O3—C19	117.38 (11)	C13—C14—H14A	119.0
C23—O4—C26	116.72 (10)	C16—C15—C14	119.42 (11)
C5—N1—C1	108.80 (8)	C16—C15—H15A	120.3
C5—N1—C11	103.25 (8)	C14—C15—H15A	120.3
C1—N1—C11	115.53 (9)	O3—C16—C15	125.09 (12)
C9—N2—C10	119.67 (10)	O3—C16—C17	115.42 (12)
C9—N2—C7	110.77 (8)	C15—C16—C17	119.49 (11)
C10—N2—C7	110.49 (8)	C18—C17—C16	120.48 (12)
N1—C1—C2	115.45 (9)	C18—C17—H17A	119.8
N1—C1—H1A	108.4	C16—C17—H17A	119.8
C2—C1—H1A	108.4	C17—C18—C13	121.44 (11)
N1—C1—H1B	108.4	C17—C18—H18A	119.3
C2—C1—H1B	108.4	C13—C18—H18A	119.3
H1A—C1—H1B	107.5	O3—C19—H19A	109.5
C12—C2—C3	115.93 (10)	O3—C19—H19B	109.5
C12—C2—C1	125.44 (10)	H19A—C19—H19B	109.5
C3—C2—C1	118.37 (9)	O3—C19—H19C	109.5
O2—C3—C2	123.17 (10)	H19A—C19—H19C	109.5
O2—C3—C4	121.64 (10)	H19B—C19—H19C	109.5
C2—C3—C4	115.15 (9)	C25—C20—C21	117.17 (10)
C3—C4—C6	114.65 (8)	C25—C20—C6	123.15 (10)
C3—C4—C5	107.25 (8)	C21—C20—C6	119.67 (10)
C6—C4—C5	117.84 (9)	C22—C21—C20	121.30 (10)
C3—C4—C10	110.70 (9)	C22—C21—H21A	119.4
C6—C4—C10	104.10 (8)	C20—C21—H21A	119.4
C5—C4—C10	101.35 (8)	C21—C22—C23	120.42 (11)
N1—C5—C4	103.40 (8)	C21—C22—H22A	119.8
N1—C5—H5A	111.1	C23—C22—H22A	119.8
C4—C5—H5A	111.1	O4—C23—C22	115.36 (10)
N1—C5—H5B	111.1	O4—C23—C24	124.82 (10)
C4—C5—H5B	111.1	C22—C23—C24	119.81 (10)
H5A—C5—H5B	109.0	C23—C24—C25	118.84 (10)
C20—C6—C4	117.40 (9)	C23—C24—H24A	120.6
C20—C6—C7	114.59 (9)	C25—C24—H24A	120.6
C4—C6—C7	102.88 (8)	C24—C25—C20	122.46 (10)
C20—C6—H6A	107.1	C24—C25—H25A	118.8
C4—C6—H6A	107.1	C20—C25—H25A	118.8
C7—C6—H6A	107.1	O4—C26—H26A	109.5
N2—C7—C6	105.66 (8)	O4—C26—H26B	109.5
N2—C7—C8	108.84 (9)	H26A—C26—H26B	109.5
C6—C7—C8	113.73 (9)	O4—C26—H26C	109.5
N2—C7—H7A	109.5	H26A—C26—H26C	109.5
C6—C7—H7A	109.5	H26B—C26—H26C	109.5
C8—C7—H7A	109.5	C28—C27—C36	118.55 (10)
C7—C8—S1	105.83 (7)	C28—C27—C10	132.36 (10)
C7—C8—H8A	110.6	C36—C27—C10	109.08 (9)
S1—C8—H8A	110.6	C27—C28—C29	119.05 (10)
C7—C8—H8B	110.6	C27—C28—H28A	120.5
S1—C8—H8B	110.6	C29—C28—H28A	120.5
H8A—C8—H8B	108.7	C30—C29—C28	122.19 (11)
N2—C9—S1	107.06 (8)	C30—C29—H29A	118.9
N2—C9—H9A	110.3	C28—C29—H29A	118.9
S1—C9—H9A	110.3	C29—C30—C31	120.14 (11)
N2—C9—H9B	110.3	C29—C30—H30A	119.9
S1—C9—H9B	110.3	C31—C30—H30A	119.9
H9A—C9—H9B	108.6	C36—C31—C32	116.57 (10)
N2—C10—C27	116.57 (9)	C36—C31—C30	116.52 (10)
N2—C10—C4	103.90 (8)	C32—C31—C30	126.90 (11)
C27—C10—C4	118.45 (9)	C33—C32—C31	120.32 (11)
N2—C10—C11	110.50 (8)	C33—C32—H32A	119.8
C27—C10—C11	103.54 (8)	C31—C32—H32A	119.8
C4—C10—C11	103.06 (8)	C32—C33—C34	122.12 (10)
O1—C11—N1	108.47 (9)	C32—C33—H33A	118.9
O1—C11—C35	112.32 (9)	C34—C33—H33A	118.9
N1—C11—C35	114.67 (9)	C35—C34—C33	118.58 (11)
O1—C11—C10	110.43 (8)	C35—C34—H34A	120.7
N1—C11—C10	105.60 (8)	C33—C34—H34A	120.7
C35—C11—C10	105.07 (8)	C34—C35—C36	119.45 (10)
C2—C12—C13	129.67 (10)	C34—C35—C11	131.91 (10)
C2—C12—H12A	115.2	C36—C35—C11	108.64 (9)
C13—C12—H12A	115.2	C31—C36—C35	122.96 (10)
C14—C13—C18	117.06 (11)	C31—C36—C27	123.53 (10)
C14—C13—C12	125.30 (11)	C35—C36—C27	113.51 (10)
C18—C13—C12	117.63 (10)		
			
C5—N1—C1—C2	47.19 (12)	C3—C2—C12—C13	172.17 (11)
C11—N1—C1—C2	−68.33 (12)	C1—C2—C12—C13	−1.9 (2)
N1—C1—C2—C12	156.00 (11)	C2—C12—C13—C14	25.1 (2)
N1—C1—C2—C3	−17.89 (14)	C2—C12—C13—C18	−153.53 (13)
C12—C2—C3—O2	20.91 (17)	C18—C13—C14—C15	2.62 (18)
C1—C2—C3—O2	−164.63 (11)	C12—C13—C14—C15	−176.01 (11)
C12—C2—C3—C4	−156.99 (10)	C13—C14—C15—C16	−0.56 (18)
C1—C2—C3—C4	17.47 (14)	C19—O3—C16—C15	−12.1 (2)
O2—C3—C4—C6	5.30 (15)	C19—O3—C16—C17	168.17 (13)
C2—C3—C4—C6	−176.76 (9)	C14—C15—C16—O3	178.80 (12)
O2—C3—C4—C5	138.19 (11)	C14—C15—C16—C17	−1.47 (19)
C2—C3—C4—C5	−43.87 (12)	O3—C16—C17—C18	−178.89 (13)
O2—C3—C4—C10	−112.08 (12)	C15—C16—C17—C18	1.3 (2)
C2—C3—C4—C10	65.86 (11)	C16—C17—C18—C13	0.8 (2)
C1—N1—C5—C4	−74.32 (10)	C14—C13—C18—C17	−2.74 (19)
C11—N1—C5—C4	48.90 (10)	C12—C13—C18—C17	175.99 (12)
C3—C4—C5—N1	72.44 (10)	C4—C6—C20—C25	77.87 (14)
C6—C4—C5—N1	−156.42 (9)	C7—C6—C20—C25	−43.01 (14)
C10—C4—C5—N1	−43.66 (10)	C4—C6—C20—C21	−103.47 (12)
C3—C4—C6—C20	75.97 (12)	C7—C6—C20—C21	135.65 (11)
C5—C4—C6—C20	−51.72 (13)	C25—C20—C21—C22	0.13 (17)
C10—C4—C6—C20	−162.95 (9)	C6—C20—C21—C22	−178.61 (11)
C3—C4—C6—C7	−157.21 (9)	C20—C21—C22—C23	0.42 (19)
C5—C4—C6—C7	75.10 (11)	C26—O4—C23—C22	179.62 (11)
C10—C4—C6—C7	−36.13 (10)	C26—O4—C23—C24	0.61 (18)
C9—N2—C7—C6	123.03 (10)	C21—C22—C23—O4	−179.83 (12)
C10—N2—C7—C6	−11.96 (11)	C21—C22—C23—C24	−0.76 (19)
C9—N2—C7—C8	0.54 (13)	O4—C23—C24—C25	179.52 (11)
C10—N2—C7—C8	−134.45 (9)	C22—C23—C24—C25	0.54 (17)
C20—C6—C7—N2	158.44 (8)	C23—C24—C25—C20	0.01 (17)
C4—C6—C7—N2	29.85 (10)	C21—C20—C25—C24	−0.34 (17)
C20—C6—C7—C8	−82.24 (11)	C6—C20—C25—C24	178.34 (10)
C4—C6—C7—C8	149.17 (9)	N2—C10—C27—C28	−60.95 (16)
N2—C7—C8—S1	29.46 (10)	C4—C10—C27—C28	64.25 (16)
C6—C7—C8—S1	−88.03 (10)	C11—C10—C27—C28	177.51 (12)
C9—S1—C8—C7	−39.36 (8)	N2—C10—C27—C36	117.99 (10)
C10—N2—C9—S1	100.24 (10)	C4—C10—C27—C36	−116.81 (10)
C7—N2—C9—S1	−30.08 (11)	C11—C10—C27—C36	−3.56 (11)
C8—S1—C9—N2	40.67 (9)	C36—C27—C28—C29	−1.56 (17)
C9—N2—C10—C27	−8.80 (14)	C10—C27—C28—C29	177.29 (12)
C7—N2—C10—C27	121.64 (10)	C27—C28—C29—C30	1.05 (19)
C9—N2—C10—C4	−141.06 (9)	C28—C29—C30—C31	0.2 (2)
C7—N2—C10—C4	−10.62 (11)	C29—C30—C31—C36	−0.86 (18)
C9—N2—C10—C11	109.00 (10)	C29—C30—C31—C32	179.89 (13)
C7—N2—C10—C11	−120.56 (9)	C36—C31—C32—C33	0.20 (18)
C3—C4—C10—N2	152.82 (8)	C30—C31—C32—C33	179.46 (12)
C6—C4—C10—N2	29.13 (10)	C31—C32—C33—C34	0.3 (2)
C5—C4—C10—N2	−93.66 (9)	C32—C33—C34—C35	−0.56 (19)
C3—C4—C10—C27	21.66 (12)	C33—C34—C35—C36	0.29 (17)
C6—C4—C10—C27	−102.03 (10)	C33—C34—C35—C11	179.41 (11)
C5—C4—C10—C27	135.18 (9)	O1—C11—C35—C34	57.75 (16)
C3—C4—C10—C11	−91.86 (9)	N1—C11—C35—C34	−66.69 (16)
C6—C4—C10—C11	144.45 (8)	C10—C11—C35—C34	177.83 (12)
C5—C4—C10—C11	21.66 (10)	O1—C11—C35—C36	−123.06 (10)
C5—N1—C11—O1	84.67 (9)	N1—C11—C35—C36	112.51 (10)
C1—N1—C11—O1	−156.69 (8)	C10—C11—C35—C36	−2.97 (11)
C5—N1—C11—C35	−148.89 (9)	C32—C31—C36—C35	−0.47 (17)
C1—N1—C11—C35	−30.25 (12)	C30—C31—C36—C35	−179.81 (11)
C5—N1—C11—C10	−33.72 (10)	C32—C31—C36—C27	179.65 (11)
C1—N1—C11—C10	84.92 (10)	C30—C31—C36—C27	0.31 (17)
N2—C10—C11—O1	−0.31 (12)	C34—C35—C36—C31	0.23 (17)
C27—C10—C11—O1	125.23 (9)	C11—C35—C36—C31	−179.08 (10)
C4—C10—C11—O1	−110.79 (9)	C34—C35—C36—C27	−179.88 (10)
N2—C10—C11—N1	116.76 (9)	C11—C35—C36—C27	0.81 (13)
C27—C10—C11—N1	−117.70 (9)	C28—C27—C36—C31	0.91 (17)
C4—C10—C11—N1	6.28 (10)	C10—C27—C36—C31	−178.19 (10)
N2—C10—C11—C35	−121.64 (9)	C28—C27—C36—C35	−178.98 (10)
C27—C10—C11—C35	3.90 (11)	C10—C27—C36—C35	1.92 (13)
C4—C10—C11—C35	127.88 (9)		

### Hydrogen-bond geometry (Å, °)

**Table:**

Cg7 and Cg9 centroids of the C20–C25 and C31–C36 rings, respectively.

**Table:**

*D*—H···*A*	*D*—H	H···*A*	*D*···*A*	*D*—H···*A*
O1—H1O1···N2	0.86 (2)	1.96 (2)	2.6253 (14)	133.3 (19)
C24—H24A···N1^i^	0.95	2.61	3.4447 (15)	146
C26—H26C···O1^i^	0.98	2.48	3.4078 (17)	157
C19—H19B···Cg7^ii^	0.98	2.82	3.4652 (18)	124
C26—H26B···Cg7^iii^	0.98	2.87	3.7720 (14)	154
C9—H9B···Cg9^iv^	0.99	2.87	3.8211 (14)	161

Symmetry codes: (i) −*x*+1, −*y*+1, −*z*+2; (ii) −*x*, −*y*+2, −*z*+2; (iii) −*x*+2, −*y*+1, −*z*+2; (iv) −*x*+1, −*y*+1, −*z*+1.
